# Computer-Assisted Colonoscopy in High–Adenoma Detection Rate Settings in a High-Risk Population: A Randomized Clinical Trial

**DOI:** 10.1001/jamanetworkopen.2026.4881

**Published:** 2026-04-15

**Authors:** Wen-Feng Hsu, Chen-Ya Kuo, Hsu-Heng Yen, Yu-Min Lin, Yen-Nien Chen, Cheuk-Kay Sun, Wei-Yuan Chang, Hsuan-Ho Lin, Hao-Yu Wu, Li-Chun Chang, Chi-Yang Chang, Ming-Shiang Wu, Han-Mo Chiu

**Affiliations:** 1 Department of Internal Medicine, National Taiwan University Hospital, Taipei; 2 Department of Internal Medicine, Fu Jen Catholic University Hospital, New Taipei, Taiwan; 3 Department of Internal Medicine, Changhua Christian Hospital, Changhua, Taiwan; 4 Division of Gastroenterology, Department of Internal Medicine, Shin Kong Wu Ho-Su Memorial Hospital, Taipei, Taiwan; 5 Department of Medicine, National Taiwan University Cancer Center, Taipei; 6 Department of Internal Medicine, National Taiwan University Hospital Hsin-Chu Branch Hsin-Chu Hospital, Hsinchu

## Abstract

**Importance:**

Computer-aided detection (CAD) systems can enhance adenoma detection, but their effectiveness in high-performance settings and among patients with positive fecal immunochemical test (FIT) results remains uncertain.

**Objective:**

To evaluate the impact of CAD on adenoma detection in routine practice, focusing on patients with positive FIT results.

**Design, Setting, and Participants:**

This multicenter, open-label, randomized clinical trial was conducted at 4 tertiary hospitals in Taiwan from February 23, 2022, to November 27, 2024. Adults aged 40 to 79 years who were scheduled for a colonoscopy owing to FIT positivity, symptoms, screening, or surveillance were randomized 1:1 to CAD-assisted or standard colonoscopy. Data were analyzed from December 1, 2024, to February 28, 2025.

**Exposures:**

Colonoscopy performed with a real-time CAD system or standard high-definition colonoscopy.

**Main Outcomes and Measures:**

The primary outcome was adenoma detection rate (ADR), defined as the proportion of patients with at least 1 histologically confirmed adenoma. Secondary outcomes included adenomas per colonoscopy (APC), sessile serrated lesion detection rate (SSLDR), and postpolypectomy surveillance intervals according to the US Multi-Society Task Force (USMSTF) and European Society of Gastrointestinal Endoscopy criteria.

**Results:**

Of 1356 randomized participants (mean [SD] age, 60.0 [9.4] years; 678 [50.0%] female and 678 [50.0%] male), CAD-assisted colonoscopy met noninferiority criteria for ADR compared with standard colonoscopy (395 of 675 [58.5%] vs 363 of 681 [53.3%]; absolute difference, 5.2 percentage points [95% CI, −0.1 to 10.5 percentage points]). Superiority was not statistically significant. CAD significantly increased mean (SD) APC (1.41 [1.95] vs 1.20 [1.88]; *P* = .01), driven mainly by detection of diminutive adenomas. In exploratory analyses of 864 patients with FIT-positive findings, CAD significantly increased ADR (288 of 441 [65.3%] vs 243 of 423 [57.4%]; *P* = .02; adjusted odds ratio [AOR], 1.39 [95% CI, 1.05-1.86]) and APC (mean [SD], 1.64 [2.08] vs 1.39 [2.09]; *P* = .01). SSLDR did not differ between groups. Consequently, CAD led to more intensive surveillance recommendations under USMSTF criteria, particularly in patients with FIT-positive findings (58 of 441 [13.2%] vs 31 of 423 [7.3%]; AOR, 1.94 [95% CI, 1.22-3.09]).

**Conclusions and Relevance:**

In this randomized clinical trial, CAD-assisted colonoscopy met noninferiority criteria for adenoma detection. Superiority was not statistically significant overall, with significant improvements limited to the exploratory FIT-positive subgroup, driven largely by diminutive adenomas. CAD also increased intensive surveillance assignments. The incremental benefit of CAD in reducing interval cancer risk requires further investigation.

**Trial Registration:**

ClinicalTrials.gov Identifier: NCT05240625

## Key Points

**Question:**

Does the use of computer-aided detection (CAD) during colonoscopy improve adenoma detection within high–adenoma detection rate (ADR) settings in the context of fecal immunochemical test (FIT)–based screening?

**Findings:**

In this randomized clinical trial of 1356 participants, CAD met noninferiority criteria for ADR. Significant increases occurred only in the exploratory FIT-positive subgroup, driven by detection of diminutive adenomas, and led to more intensive surveillance intervals based on US Multi-Society Task Force guidelines.

**Meaning:**

CAD-assisted colonoscopy was noninferior to standard colonoscopy; however, detection gains involved mainly small lesions and increased intensive surveillance recommendations, while long-term clinical benefits remain uncertain.

## Teaser

This randomized clinical trial evaluates the efficacy of computer-aided detection–assisted vs standard colonoscopy in routine practice among patients with a high risk for colorectal cancer.

## Introduction

Colorectal cancer (CRC) is the third most common cancer and the second leading cause of cancer death worldwide.^1^ Screening reduces the incidence and mortality of CRC.^2,3^ The success of any screening strategy, whether the fecal immunochemical test (FIT) or direct screening colonoscopy, relies on high-quality colonoscopy to remove precancerous lesions.^4,5^

The effectiveness of colonoscopy in preventing CRC depends largely on the quality, commonly measured by the adenoma detection rate (ADR).^6^ The ADR is inversely correlated with postcolonoscopy CRC (PCCRC); each 1% increase in ADR reduces interval CRC risk by 3%.^7,8^ Consequently, professional societies established ADR benchmarks of 35% or greater for high-quality practice.^9^

Despite its efficacy, colonoscopy remains operator dependent, with miss rates as great as 26% for adenomas and 27% for serrated polyps.^10^ This diagnostic gap is a primary contributor to the occurrence of PCCRC. Missed lesions are typically diminutive (≤5 mm) or small (6-9 mm), flat or nonpolypoid, and often located in the proximal colon.^10,11^ Artificial intelligence (AI)–based computer-aided detection (CAD) systems were developed to overcome these limitations. Numerous trials have shown that CAD increases ADR and detection of adenomas per colonoscopy (APC).^12,13^ However, its impact on the detection of advanced adenomas is less evident than that for diminutive or small adenomas.^14^ Accordingly, the American Gastroenterological Association neither recommends nor discourages routine CAD use, citing uncertain long-term benefits and potential resource strain from removing low-risk polyps.^14^

The limited effect of CAD on advanced lesions raises questions about its value in populations with high baseline ADR, such as patients with FIT-positive findings, whose ADRs typically range from 47% to 67%.^15,16^ Therefore, the efficacy of CAD among the specific high-risk population and in high ADR settings remains a crucial area for investigation. This multicenter randomized clinical trial (RCT) evaluated the efficacy of CAD-assisted vs standard colonoscopy in routine practice at 4 high-performance centers, specifically focusing on high-risk patients with FIT-positive findings.

## Methods

### Study Design and Participants

This prospective, multicenter RCT was conducted at 4 high-performance centers in Taiwan (National Taiwan University Hospital, Taipei; Fu Jen Catholic University Hospital, New Taipei; Changhua Christian Hospital, Changhua; and Shin Kong Wu Ho-Su Memorial Hospital, Taipei) between February 23, 2022, and November 27, 2024. These 4 centers had high ADRs in the national program’s screening database between 2019 and 2021 (mean [SD] ADR, 56.1% [1.6%] for National Taiwan University Hospital, 56.6% [2.3%] for Fu Jen Catholic University Hospital, 51.4% [1.8%] for Changhua Christian Hospital, and 70.7% [2.5%] for Shin Kong Wu Ho-Su Memorial Hospital). The study protocol (Supplement 1) was approved by the Institutional Review Board at each center. All participants provided written informed consent. We followed the Consolidated Standards of Reporting Trials (CONSORT) reporting guideline and its extension for AI interventions.

Eligible participants were aged 40 to 79 years and were undergoing colonoscopy for positive FIT results, gastrointestinal symptoms, screening, or surveillance for a history of polyps. Exclusion criteria included a history of CRC, polyposis syndromes, inflammatory bowel disease, prior colorectal surgery, inadequate bowel preparation, or incomplete colonoscopy.

### Randomization and Interventions

**Figure 1.**
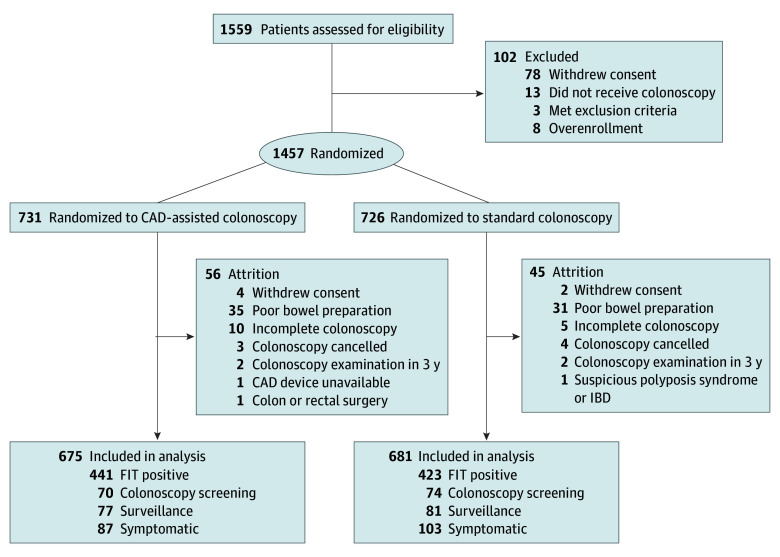
Study Flow Diagram CAD indicates computer-aided detection; FIT, fecal immunochemical test; and IBD, inflammatory bowel disease.

Participants were randomized 1:1 to CAD-assisted colonoscopy or standard high-definition colonoscopy using a web-based computer-generated randomization sequence. Recruitment staff, participants, and endoscopists (W.F.H., C.Y.K., H.H.Y., Y.M.L., Y.N.C., C.K.S., W.Y.C., H.H.L., H.Y.W., L.C.C., C.Y.C., and H.M.C.) were aware of the group allocation due to the nature of the intervention, while histopathologists were blinded. Procedures were performed by board-certified endoscopists (including W.F.H., C.Y.K., H.H.Y., Y.M.L., Y.N.C., C.K.S., W.Y.C., H.H.L., H.Y.W., L.C.C., C.Y.C., and H.M.C.), classified as senior (≥2000 lifetime procedures) or junior (<2000 procedures) (Figure 1).

Standard colonoscopy used standard high-definition colonoscopes (EVIS LUCERA Elite System Olympus CV-290 or CLV-290SL [Olympus Corporation] and ELUXEO 7000 series EC-760R or EC-760ZP [Fujifilm Co]) with a minimum withdrawal time of 6 minutes for negative procedures. In the CAD-assisted colonoscopy group, procedures were performed using the same high-definition colonoscopes connected to a real-time AI-based CAD system (aetherAI Endo [aetherAI Co Ltd]). The system alerts the endoscopist to suspected polyps with an auditory signal and a blue bounding box on a secondary monitor. Detailed algorithm architecture and training datasets are provided in eMethods 1 in Supplement 2.

### Procedures and Outcomes

Bowel preparation was graded by the endoscopist using the Aronchick Scale (poor, fair, good, or excellent); participants with inadequate preparation, defined as a rating of poor, were excluded.^17^ Endoscopists recorded the size, location (proximal colon from the cecum to the splenic flexure or distal colon from the descending colon to the rectum), and morphology according to the Paris classification. All detected polyps were resected using standard techniques and reviewed by board-certified gastrointestinal pathologists blinded to the study group assignment.

The primary outcome was ADR, defined as the proportion of patients with at least 1 histologically confirmed adenoma. The primary hypothesis was that CAD-assisted colonoscopy would be noninferior to standard colonoscopy regarding ADR. Secondary outcomes included (1) APC, (2) polyps per colonoscopy, (3) advanced APC, (4) nonneoplastic polypectomy rate, (5) sessile serrated lesion detection rate (SSLDR), and (6) withdrawal time. Detailed definitions of these secondary outcomes are provided in eMethods 2 in Supplement 2.

We also evaluated the recommended postpolypectomy surveillance interval according to the guidelines of the US Multi-Society Task Force (USMSTF) and the European Society of Gastrointestinal Endoscopy (ESGE).^18,19^ We compared the proportion of patients assigned to intensive surveillance intervals, defined as 3 to 5 years or 3 years under USMSTF criteria and 3 years under ESGE criteria, between the 2 groups.

### Statistical Analysis

The study was designed to test noninferiority, followed by a conditional test for superiority. The sample size calculation was driven by the superiority assumption to ensure sufficient power. Assuming a baseline ADR of 48% in the standard colonoscopy group, we aimed to detect an absolute increase of 8.4% in the CAD-assisted colonoscopy group based on previous meta-analyses demonstrating a relative increase with CAD.^12,13^ With 90% power and a 2-sided α = .05, 1480 participants (740 per group) were required. Consequently, this sample size provided more than 99% power to demonstrate noninferiority with a prespecified margin of −10%. Accounting for a 5% dropout rate, the final target was 1560 participants (780 per group).

Data were analyzed from December 1, 2024, to February 28, 2025. Analyses were performed on an intention-to-treat basis. For baseline characteristics, standardized mean differences (SMDs) were calculated to assess group balance. For the primary outcome of ADR, noninferiority was established if the 95% CI lower bound for the risk difference exceeded −10 percentage points, followed by conditional superiority testing. For secondary outcomes, continuous variables were compared using the unpaired *t* test or Mann-Whitney test, as appropriate. Categorical variables were compared using the χ^2^ test or Fisher exact test. Multivariable logistic regression was used to calculate adjusted odds ratios (AORs) and 95% CIs for factors associated with adenoma detection, adjusting for the prespecified stratification variables (age, sex, indication, endoscopist experience, and participating center). For count data, such as APC, negative binomial regression models were used to calculate adjusted incidence rate ratios (AIRRs) and 95% CIs. A 2-sided *P* < .05 was considered statistically significant. To assess potential contamination bias or learning effects, we compared the ADR in the standard colonoscopy group between the first and second halves of the enrollment period. Statistical analyses were performed using SAS software, version 9.4 (SAS Institute Inc).

## Results

### Participant Characteristics

A total of 1356 participants (mean [SD] age, 60.0 [9.4] years; 678 [50.0%] female and 678 [50.0%] male) were included in the final analysis. Among 1559 patients initially assessed for eligibility, 1457 were randomized: 731 to CAD-assisted colonoscopy and 726 to standard colonoscopy. After exclusions, the final analysis included 675 patients in the CAD group and 681 in the standard group (Figure 1).

Baseline characteristics were well balanced, with SMDs less than 0.10 for all major variables (Table 1). The mean (SD) age was 60.0 (9.3) years in the CAD-assisted group and 60.0 (9.5) in the standard group, and the sex distribution was similar. The most common indication for colonoscopy was a positive FIT result, accounting for 441 of 675 patients (65.3%) in the CAD group and 423 of 681 (62.1%) in the standard group, followed by symptomatic evaluation, surveillance, and screening. Endoscopist experience was comparable between groups, with senior endoscopists (≥2000 lifetime procedures) performing 463 (68.6%) CAD colonoscopies and 489 (71.8%) standard colonoscopies.

**Table 1.** Baseline Demographics and Clinical Characteristics of Study Participants

Characteristic	Colonoscopy type, No. (%)	
	Standard (n = 681)	CAD-assisted (n = 675)
Age, mean (SD), y	60.0 (9.5)	60.0 (9.3)
Sex		
Female	345 (50.7)	333 (49.3)
Male	336 (49.3)	342 (50.7)
Indication for colonoscopy		
FIT-positive	423 (62.1)	441 (65.3)
Symptomatic	103 (15.1)	87 (12.9)
Screening	74 (10.9)	70 (10.4)
Surveillance	81 (11.9)	77 (11.4)
Endoscopist experience		
Senior (≥2000 procedures)	489 (71.8)	463 (68.6)
Junior (<2000 procedures)	192 (28.2)	212 (31.4)

Abbreviations: CAD, computer-aided detection; FIT, fecal immunochemical test.

### Primary Outcome

**Figure 2.**
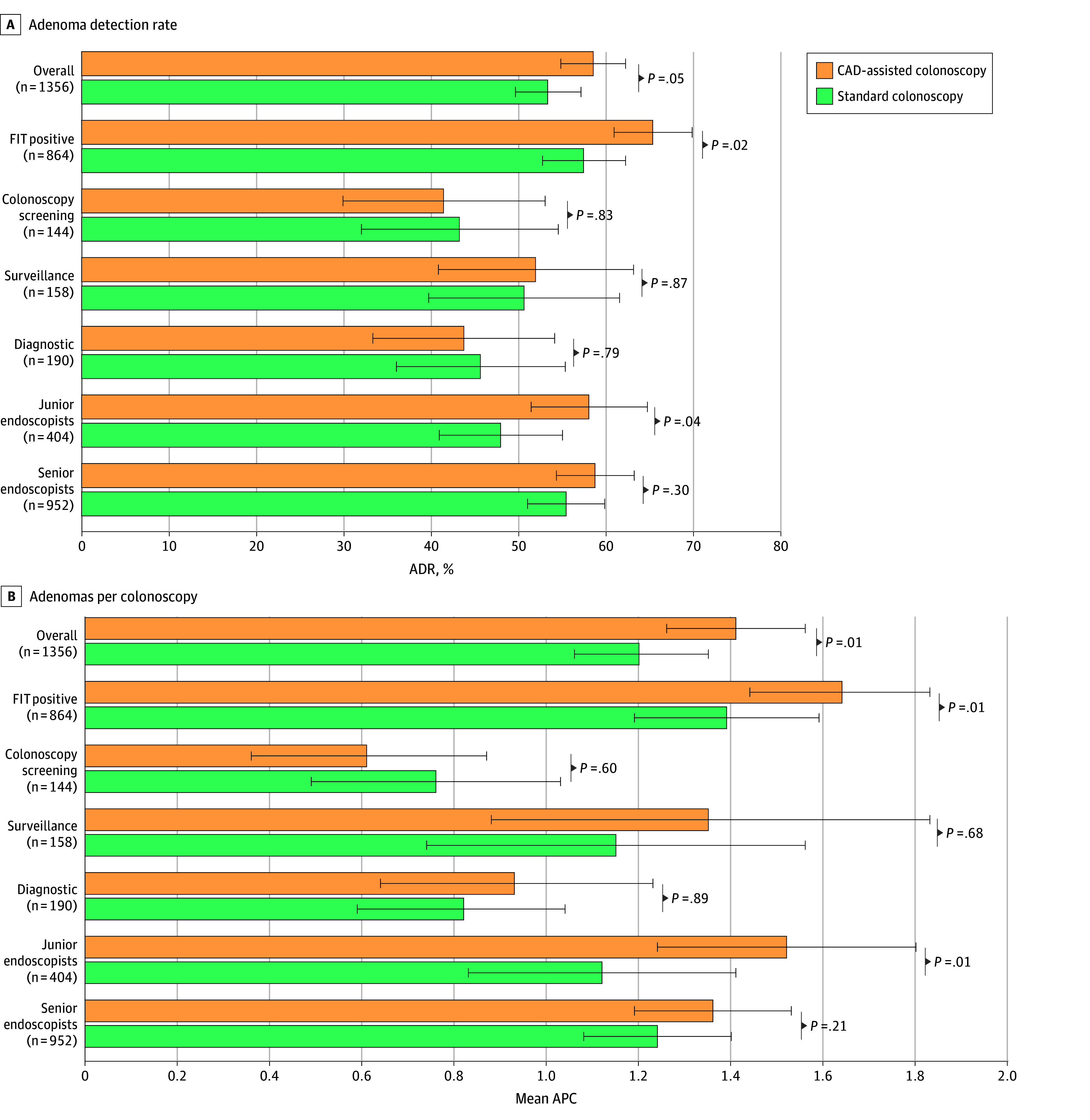
Bar Graphs Showing Comparison of Adenoma Detection Rate (ADR) and Number of Adenomas Per Colonoscopy (APC) Between Computer-Aided Detection (CAD)–Assisted and Standard Colonoscopy Across Subgroups FIT indicates fecal immunochemical test. Error bars indicate 95% CIs.

The primary noninferiority end point for ADR was met (Table 2). The ADR was 58.5% (395 of 675 patients) in the CAD-assisted colonoscopy group and 53.3% (363 of 681 patients) in the standard colonoscopy group (absolute difference, 5.2 percentage points [95% CI, −0.1 to 10.5 percentage points]; *P* = .01 for noninferiority). In the subsequent analysis for superiority, the ADR was numerically higher in the CAD group but was not statistically significant (395 of 675 [58.5%] vs 363 of 681 [53.3%]; *P* = .05) (Figure 2A).

**Table 2.** Primary and Secondary Outcomes in the Intention-to-Treat Population

Outcome	Colonoscopy type		Difference (95% CI)	*P* value^a^
	Standard (n = 681)	CAD assisted (n = 675)		
Primary outcome				
ADR, No. (%)	363 (53.3)	395 (58.5)	5.2 (−0.1 to 10.5) pp	.001
Secondary outcomes				
APC, mean (SD), No.	1.20 (1.88)	1.41 (1.95)	0.21 (0.01 to 0.41)	.01
PPC, mean (SD), No.	1.71 (2.28)	1.97 (2.33)	0.27 (0.02 to 0.51)	.01
Advanced APC, mean (SD), No.	0.24 (0.53)	0.26 (0.58)	0.02 (−0.04 to 0.08)	.89
NNPR, No. (%)	28 (4.1)	31 (4.6)	0.5 (−1.7 to 2.7) pp	.66
SSLDR, No. (%)	33 (4.8)	29 (4.3)	−0.6 (−2.8 to 1.7) pp	.63
Withdrawal time, mean (SD), min	8.32 (4.01)	9.14 (4.09)	0.82 (0.39 to 1.25)	<.001

Abbreviations: ADR, adenoma detection rate; APC, adenomas per colonoscopy; CAD, computer-aided detection; NNPR, nonneoplastic polypectomy rate; pp, percentage points; PPC, polyps per colonoscopy; SSLDR, sessile serrated lesion detection rate.

^a^ Primary outcome measured using *P* value for noninferiority. Secondary outcomes measured using *P* value for superiority (*P* < .05 indicates statistical significance).

### Secondary Outcomes

The mean (SD) APC was significantly higher for CAD compared with standard colonoscopy (1.41 [1.95] vs 1.20 [1.88]; *P* = .01) (Table 2). Similarly, the mean (SD) number of polyps per colonoscopy was significantly higher in the CAD group (1.97 [2.33] vs 1.71 [2.28]; *P* = .005). In terms of advanced pathology, there was no significant difference in the mean (SD) number of advanced APC between groups (0.26 [0.58] vs 0.24 [0.53]; *P* = .89). Importantly, the nonneoplastic polypectomy rate did not differ significantly between the CAD and standard groups (31 of 675 [4.6%] vs 28 of 681 [4.1%]; *P* = .66), indicating that CAD did not lead to a surge in unnecessary polypectomies. Overall, SSLDR did not differ significantly between groups (29 of 675 [4.3%] vs 33 of 681 [4.8%]; *P* = .63), a null finding that was consistent across all subgroups (eTable 1 in Supplement 2). Finally, the mean (SD) withdrawal time was slightly longer in the CAD group compared with the standard group (9.14 [4.09] vs 8.32 [4.01] minutes; *P* < .001). No major adverse events occurred in either group.

### Exploratory Subgroup Analyses

In exploratory analyses stratified by indication, the benefit of CAD appeared most pronounced in the FIT-positive subgroup, where ADR was significantly higher in the CAD group than in the standard group (288 of 441 [65.3%] vs 243 of 423 [57.4%]; *P* = .02). In contrast, no significant differences were observed in the screening, surveillance, or symptomatic subgroups (Figure 2A). Given the significant difference in the FIT-positive subgroup, we performed multivariable logistic regression (eTable 2 in Supplement 2). After adjustment for age, sex, and participating center, CAD-assisted colonoscopy remained independently associated with a higher likelihood of adenoma detection (AOR, 1.39 [95% CI, 1.05-1.86]).

The benefit of APC was also consistent in the FIT-positive subgroup (mean [SD], 1.64 [2.08] vs 1.39 [2.09]; *P* = .01) (Figure 2B). Multivariable negative binomial regression showed a trend toward a higher adenoma yield in the overall population (AIRR, 1.16 [95% CI, 1.00-1.34]) and specifically in patients with positive FIT results (AIRR, 1.18 [95% CI, 1.00-1.38]) (eFigure in Supplement 2).

When stratified by endoscopist experience, the benefit of CAD was most evident among junior endoscopists, who achieved a significantly higher ADR (123 of 212 [58.0%] vs 92 of 192 [47.9%]; *P* = .04) and APC (mean [SD], 1.52 [2.09] vs 1.12 [2.00]; *P* = .01) with CAD assistance (Figure 2). Among senior endoscopists, ADR and APC were numerically higher with CAD but did not reach statistical significance.

Last, we evaluated temporal trends to rule out learning effects. The ADR in the standard colonoscopy group remained stable between the first and second halves of the study period (180 of 340 [52.9%] vs 183 of 341 [53.7%]; *P* = .34), suggesting no significant contamination bias (eTable 3 in Supplement 2).

### Characteristics of Detection Lesions

In the overall population, the increase in adenoma yield with CAD was mainly driven by diminutive adenomas, with a significantly higher mean (SD) number per colonoscopy compared with standard colonoscopy (0.91 [1.46] vs 0.75 [1.27]), corresponding to an AIRR of 1.22 (95% CI, 1.03-1.43), without significant differences for adenomas measuring 6 to 9 mm or 10 mm or greater (eTable 4 in Supplement 2) By location, CAD significantly improved proximal adenoma detection (AIRR, 1.20 [95% CI, 1.01-1.44]) but not distal adenoma detection. Regarding morphology, detection was numerically higher for both types, with a trend toward increased yield for nonpolypoid adenomas (AIRR, 1.22 [95% CI, 0.99-1.49]).

In the FIT-positive subgroup, similar trends were observed but with stronger effects (eTable 5 in Supplement 2). CAD significantly increased the detection of diminutive adenomas (AIRR, 1.26 [95% CI, 1.03-1.53]), but not adenomas 6 mm or greater. CAD showed a numerical increase in proximal (mean [SD], 0.86 [1.50] vs 0.74 [1.44]; *P* = .18) and nonpolypoid adenomas (mean [SD], 0.76 [1.41] vs 0.64 [1.32]; *P* = .12), but the differences were nonsignificant.

### The Impact of Surveillance Interval

Patients in the CAD group were more frequently assigned to USMSTF intensive surveillance of 3 to 5 years compared with the standard group (70 of 675 [10.4%] vs 49 of 681 [7.2%]; AOR, 1.50 [95% CI, 1.01-2.21]) (Table 3). This effect was more pronounced in the FIT-positive subgroup (58 of 441 [13.2%] vs 31 of 423 ]7.3%]; AOR, 1.94 [95% CI, 1.22-3.09]). Conversely, no significant differences were observed for stricter 3-year intervals under either USMSTF or ESGE criteria in either population.

**Table 3.** Difference in the Proportion of Surveillance Intervals Between Study Groups

Surveillance (interval)	Colonoscopy type, No./total No. (%)		CAD-assisted vs standard colonoscopy		
	CAD-assisted	Standard	Proportion difference (95% CI), pp	AOR (95% CI)^a^	*P* value
**Overall cases**					
USMSTF intensive (3-5 y)	70/675 (10.4)	49/681 (7.2)	3.2 (0.2 to 6.2)	1.50 (1.01-2.21)	.04
USMSTF intensive (3 y)	142/675 (21.0)	134/681 (19.7)	1.4 (−3.0 to 5.7)	1.09 (0.83-1.43)	.54
ESGE intensive (3 y)	124/675 (18.4)	122/681 (17.9)	0.5 (−3.7 to 4.6)	1.04 (0.78-1.37)	.81
**FIT-positive cases**					
USMSTF intensive (3-5 y)	58/441 (13.2)	31/423 (7.3)	5.8 (1.8 to 9.8)	1.94 (1.22-3.09)	.01
USMSTF intensive (3 y)	111/441 (25.2)	97/423 (22.9)	2.2 (−3.5 to 7.9)	1.17 (0.85-1.61)	.34
ESGE intensive (3 y)	98/441 (22.2)	88/423 (20.8)	1.4 (−4.1 to 6.9)	1.13 (0.81-1.58)	.46

Abbreviations: AOR, adjusted odds ratio; CAD, computer-aided detection; ESGE, European Society of Gastrointestinal Endoscopy; FIT, fecal immunochemical test; pp, percentage points; USMSTF, the US Multi-Society Task Force.

^a^ Adjusted for the prespecified stratification variables (age, sex, indication, endoscopist experience, and participating center).

## Discussion

This multicenter RCT demonstrated that CAD-assisted colonoscopy was noninferior to standard colonoscopy for ADR in a high-ADR setting. While the ADR was numerically higher with CAD, it did not reach statistical superiority; however, APC was increased. The benefit was most evident in our exploratory analysis of patients with FIT-positive findings, showing an absolute ADR increase of 7.9%. CAD also provided measurable benefit for junior endoscopists, suggesting potential to reduce performance gaps related to operator experience. However, the observed increase in adenoma yield was primarily driven by diminutive adenomas, while the detection of advanced adenomas was comparable between groups. Consequently, these findings suggest that while CAD may not significantly alter the detection of advanced pathology in high-performing centers, it serves as a valuable tool for maximizing overall adenoma capture, particularly in high-risk cohorts and for reducing performance variability among endoscopists.

Our findings align with those of previous meta-analyses^12,13^ showing that CAD systems improve both ADR and APC. Consistent with earlier reports, the incremental yield was mainly from diminutive adenomas.^20,21^ We also observed a trend toward increased proximal adenoma detection with CAD, consistent with prior studies.^12,20,21^ Although CAD enhances the identification of subtle lesions, its clinical significance is uncertain given the lack of benefit for advanced adenomas.^12,13,20,21^ In particular, whether the only additional detection of diminutive and proximal adenomas meaningfully reduces the risk of PCCRC remains unclear.^14^ Moreover, we found that CAD use was associated with a shift toward shorter surveillance intervals under USMSTF recommendations, especially among patients with positive FIT results, raising questions about the balance between improved detection and downstream resource utilization.

Equally important is our null finding regarding SSLDR. In our study, CAD did not significantly improve SSLDR compared with standard colonoscopy. This result should be interpreted with caution. The relatively low prevalence of sensile serrated lesions in our cohort (approximately 4%) likely limited statistical power, and most participating endoscopists were highly experienced, which may have contributed to high baseline SSLDR detection and attenuated the incremental benefit of CAD. Prior studies^21,22,23,24^ have also shown inconsistent effects of CAD on sensile serrated lesion detection, with some reporting modest improvements while others have found no significant difference. Taken together, our null finding may reflect sample size constraints and the high performance of experienced endoscopists, rather than a true limitation of CAD technology.

Concerns regarding unnecessary resections of nonneoplastic lesions were addressed in our study. We found no significant difference in the nonneoplastic resection rate, suggesting that endoscopists effectively discriminated between false-positive alerts and true lesions. While withdrawal time was prolonged by approximately 0.8 minutes in the CAD group, this modest increase is likely a trade-off for the higher adenoma yield and is clinically acceptable.

The broader discourse on AI in endoscopy includes important considerations about its potential downside. While our study demonstrates a clear benefit, it is important to acknowledge the ongoing debate. Some studies^25,26^ have reported limited or no benefit to CAD, particularly among expert endoscopists with very high baseline ADRs, suggesting a potential ceiling effect. A recent observational study^27^ has raised concern about “deskilling,” whereby reliance on AI could erode endoscopists’ intrinsic detection skills over time. Our findings offer a nuanced perspective. In the FIT-positive subgroup, multivariable analysis showed that endoscopist experience was not a significant determinant of detection. This likely reflects the high rates of adenoma prevalence creating a target-rich environment that attenuates operator variability, combined with a standardization effect where CAD serves as a constant second observer to narrow the performance gap between junior and senior endoscopists. These findings suggest that implementation of CAD in organized screening programs may help ensure uniformly high-quality performance, regardless of the endoscopist’s years of practice.

### Strengths and Limitations

The principal strength of this study lies in its robust design as a large, prospective, multicenter RCT, which enhances both the internal validity and external generalizability of our findings. By including a diverse group of endoscopists with varying levels of experience from both academic tertiary hospitals and community-based centers, the results are highly representative of clinical practice rather than limited to expert settings. This heterogeneity also allowed us to explore the impact of CAD across different operator experience levels, providing novel insights into its potential role in reducing performance variability. Another important strength is the deliberate focus on individuals with FIT-positive findings as the primary analysis population. This group represents a clinically relevant, high-yield cohort in organized CRC screening programs, where both ADR and APC are substantially higher than in average-risk screening. Evaluating CAD in this context not only addresses a critical knowledge gap but also provides evidence directly applicable to population-based screening strategies. Furthermore, the comprehensive assessment of outcomes—including ADR, APC, lesion characteristics, and downstream surveillance interval recommendations—offers a more holistic evaluation of CAD’s clinical impact than most previous RCTs.

Several limitations must also be acknowledged. First, due to the nature of the intervention, it was not possible to blind the endoscopists to the study arm, which may have introduced performance bias or the Hawthorne effect.^28^ However, the consistent benefit observed across different centers and experience levels suggests this was not a major factor. Second, this trial evaluated a single CAD system; therefore, the results may not be generalizable to all AI platforms, as performance can vary based on the underlying algorithms and training datasets. Third, the impact of increased detection of diminutive adenomas is unclear. A previous study in the FIT screening program reported that ADR was inversely associated with PCCRC risk, but the risk did not differ significantly among the upper 3 ADR tiers.^29^ Moreover, the study was not powered to assess long-term outcomes, such as PCCRC. Long-term follow-up studies of cohorts from large RCTs are needed to definitively determine whether the CAD improves PCCRC prevention. Fourth, although the same endoscopists performed procedures in both arms, raising the possibility of a learning effect, our temporal analysis showed no significant increase in ADR in the standard arm over time. This suggests that contamination bias was minimal and unlikely to have masked a true benefit of CAD.

## Conclusions

In this RCT, a clinical CAD system was noninferior to standard colonoscopy for ADR and improved APC, primarily driven by detection of diminutive adenomas, in a high-risk, FIT-positive population. Further research is needed to demonstrate the effectiveness of CAD in reducing CRC incidence before it can become the standard of care in colonoscopy practice and be implemented in population-based screening programs.

## Notes

Supplement 1.
Trial Protocol

Supplement 2. **eMethods 1.** Detailed Description of the AI System **eMethods 2.** Definitions of Secondary End Points **eFigure.** Adjusted IRR for Adenomas per Colonoscopy With CAD vs Standard Colonoscopy **eTable 1.** Comparison of the Sessile Serrated Lesion Detection Rate (SSLDR) Between CAD-Assisted and Standard Colonoscopy **eTable 2.** Regression Analyses for Factors Associated With Adenoma Detection Rate (ADR) for FIT-Positive Colonoscopy **eTable 3.** Comparison of Adenoma Detection Rate in the Standard Colonoscopy Group Between the First and Second Halves of the Study Period **eTable 4.** Comparison of Adenomas Detected per Colonoscopy by Lesion Characteristics Between CAD-Assisted and Standard Colonoscopy **eTable 5.** Comparison of Adenomas Detected per Colonoscopy Among FIT-Positive Patients: CAD-Assisted vs Standard Colonoscopy

Supplement 3.
Data Sharing Statement
