# Error in Abstract

**DOI:** 10.1001/jamanetworkopen.2026.17639

**Published:** 2026-05-15

In the Original Investigation titled “Computer-Assisted Colonoscopy in High–Adenoma Detection Rate Settings in a High-Risk Population: A Randomized Clinical Trial,” published April 15, 2026, the trial registration number was incorrect in the Abstract. This article has been corrected.^1^
