# Supplementary material for: Computer-Assisted Colonoscopy in High–Adenoma Detection Rate Settings in a High-Risk Population: A Randomized Clinical Trial
Source: JAMA Netw Open. 2026 Apr 15;9(4):e264881. doi: 10.1001/jamanetworkopen.2026.4881 (PMC13084460; doi:10.1001/jamanetworkopen.2026.4881)
Supplement: Supplement 1. — Trial Protocol [file jamanetwopen-e264881-s001.pdf]

1. Cover Page

**Study Title**	Clinical efficacy evaluation of a computer-aided colonoscopy as compared with the standard colonoscopy.
**Protocol Number**	EndoRCT2021
**Protocol Version**	v1.2
**Study Type**	An interventional study
**Investigational Sites**	(1) National Taiwan University Hospital<br>(2) Fu Jen Catholic University Hospital
**Principal Investigator**	Han-Mo Chiu<br>Attending physician | Department of Internal Medicine, National Taiwan University Hospital
**Co-Investigators**	Chi-Yang Chang<br>Deputy Superintendent | Fu Jen Catholic University Hospital
**Investigational Device**	
Device Name	"aetherAI" Computer-aided Polyp Detection (CADe) Systems for Colonoscopy
Device Version	Version: 100001
Device Class	Class 2
Device Function	Intended to automatically detect potential polyps during colonoscopy. The identified polyps can be highlighted to the endoscopists during real-time colonoscopy procedures.
**Manufacturer**	aetherAI Co., Ltd.
**Address**	9F, No. 3-2, Park Street, Nangang District, Taipei, 115, Taiwan
**Telephone**	886-2-27856892
**Protocol Author**	Cathy Kao, MSc.<br>Medical writer, aetherAI Co., Ltd.
**Date of Initial Document**	November 18, 2021

2. Table of Contents

1. **COVER PAGE** 1

2. **TABLE OF CONTENTS** 2

3. **Protocol Synopsis** 4

4. **DEVICE DESCRIPTION** 6
- OVERALL DESCRIPTION 6
- INTENDED USE 6
- CONTRAINDICATIONS 6
- ANALYTICAL PERFORMANCE 6
- MARKET ACCESS 6

5. **STUDY RATIONALE** 7

6. **OBJECTIVES** 8

7. **STUDY DESIGN** 9
- STUDY STRUCTURE 9
- STUDY POPULATION 9
  - *Inclusion criteria* 9
  - *Exclusion criteria* 9
- STUDY PROCEDURES 9
  - *Screening (Screening Visit)* 9
  - *Enrollment* 10
  - *Randomization* 10
  - *Blinding* 10
  - *Colonoscopy Procedures (Intervention Visit)* 10
  - *Histopathology* 11
  - *Withdrawal of Subjects* 11
- ENDPOINTS 11
  - *Primary Endpoint* 11
  - *Secondary Endpoints* 12
- DATA COLLECTION 13

8. **STATISTICAL ANALYSIS** 13
- SAMPLE SIZE ESTIMATION 14
- ANALYSIS POPULATION 14
- STATISTICAL METHODS 14

9. **RISKS AND BENEFITS** 16

10. ETHICAL COMPLIANCE 17

11. REFERENCES 18

12. APPENDICES 19

BOWEL PREPARATION SCALE (HSU ET AL., 2016) 19

PARIS CLASSIFICATION (ENDOSCOPIC CLASSIFICATION REVIEW, 2005) 19

3. Protocol Synopsis

**Study Title**	Clinical efficacy evaluation of a computer-aided colonoscopy as compared with the standard colonoscopy.
**Study Design**	A prospective, multi-center, parallel-assigned, two-arm, randomized controlled trial
**Study Objective(s)**	To evaluate the performance of a computer-aided detection device that is expected to result in non-inferior or improved adenoma detection rate (ADR) when compared with the current standard-of-care procedure.
**Study Population**	The target population includes individuals who are undergoing screening, diagnostic, or surveillance colonoscopy.<br>Main inclusion criteria:<br>(1) Age of ≥ 40 years and < 80 years<br>(2) Subjects who have given signed informed consent form<br>(3) Informed consensus has been obtained that endoscopic resection should be performed if a lesion is found<br>(4) Subjects who are scheduled for screening or diagnostic colonoscopy for colorectal cancer (CRC) or surveillance colonoscopy for post-polypectomy follow-up
**Study Endpoints**	Primary endpoint:<br>● ADR<br>Secondary endpoints:<br>● Polyp detection rate (PDR)<br>● Adenomas per colonoscopy (APC)<br>● Polyps per colonoscopy (PPC)<br>● Non-neoplastic polypectomy rate (NNPR)<br>● Sessile serrated lesions per colonoscopy (SPC)<br>● Advanced adenomas per colonoscopy (AAPC)<br>● Withdrawal time
**Duration of Study**	The study will include a Screening Visit (1 day) and an Intervention Visit (1 day) for colonoscopy.

**Statistical Analysis**	The primary analysis will be the comparison of ADR between an AI-aided colonoscopy and the standard colonoscopy. Differences in ADR between two arms will be expressed as relative risk (RR) with 95% CIs. Non-inferiority will be met for the primary endpoint (ADR) if the lower 2-sided 95% confidence interval (CI) excludes a 10% or greater difference in favor of the control group. The 10% non-inferiority margin reflects a typical maximum clinically acceptable difference for comparative studies. If non-inferiority is demonstrated for the primary endpoint, the endpoint will be assessed for superiority (one-sided p value <0.025) using the Fisher exact test.
**Sample Size**	The sample size is calculated based on the evaluation of primary outcome ADR. Based on the results of meta-analysis, an expected ADR ranges from around 20% to 40% for computer-aided colonoscopy and SC. A sample size ranging from 198 to 297 per arm is required to establish non-inferiority with a non-inferiority margin of 10%, power of 80% and an alpha level of 2.5% (one-sided). Considering a withdrawal rate of 10% due to potential exclusions and drop-outs, the enrollment goal is 220 to 330 subjects per arm.<br><br>However, interim analysis of this study performed in October 2022 showed that the ADR in our SC group is 43%, which is significantly higher than the ADR results acquired from meta-analysis (around 20%); therefore, the enrollment goal is increased to 400 subjects per arm.

4. Device Description

4.1. Overall Description

The investigational medical device is intended to automatically detect potential polyps via colonoscopy in real-time during colonoscopy examinations.
The subject device contains an artificial intelligence/machine learning (AI/ML) advanced algorithm to aid the endoscopists in detection of colonic mucosal lesions and the detected polyps will be highlighted to the endoscopists during the real-time colonoscopy procedures.

4.2. Intended Use

The investigational medical device is intended to be used by qualified endoscopists during colonoscopic examinations to aid detection of colonic mucosal lesions. The target population is adults aged at least 20 years, but not those with a history of colorectal surgeries, with a contraindication to colonoscopy, or pregnancy.

4.3. Contraindications

(1) Pregnant patients.
(2) Patients with lower gastrointestinal hemorrhage.
(3) Patients who had received colorectal surgeries.

4.4. Analytical Performance

The standalone algorithm testing has demonstrated that the investigational medical device could achieve a recall of 93.3% and a precision of 92.6% in poly detection using 9098 images from two study sites (Cathay General Hospital in Taiwan: 3122 images; National Taiwan University Hospital: 5976 images) in Taiwan.

4.5. Market Access

The investigational medical device has been submitted to the local health authority (Taiwan FDA) in May 2021 for market approval and is currently under review.

5. Study Rationale

Colonoscopy is clinically used as the gold standard for detection of colorectal cancer (CRC) and removal of adenomatous polyps of the colon and rectum. Evidence has shown that CRC could be prevented by colonoscopic removal of adenomatous polyps. Despite the success of colonoscopy in reducing cancer-related deaths, there exists a disappointing level of adenomas missed at colonoscopy (Shaukat et al., 2021). In recent years, emerging artificial intelligence (AI) and computer-aided detection (CADe) technology has been shown to improve ADR (Barua et al., 2021; Hassan et al., 2021; Mohan et al., 2020; Shaukat et al., 2021; Spadaccini et al., 2021). Based on a meta-analysis, ADR was demonstrated to be significantly higher in the CADe groups than in the standard colonoscopy groups, representing a relative risk of 25.2%.

In this study, performance of colonoscopy with or without aid of CADe will be compared in terms of quality indicators. The adenoma detection rate (ADR), which is the proportion of average-risk patients undergoing screening colonoscopy in whom an adenoma is found, is regarded as a robust measure of colonoscopy performance quality that correlates with subsequent cancer risk (Shaukat et al., 2021). Thus, ADR is taken as the primary outcome of this study. The target population includes individuals who are undergoing screening, diagnostic, or surveillance colonoscopy.

6. Objectives

The goal of this study is to evaluate the performance of a CADe device that is expected to result in non-inferior or improved ADR when compared with the current standard-of-care procedure.
The main objective of this study is to compare the ADR between a computer-aided colonoscopy (CC) and standard colonoscopy (SC) in human subjects undergoing screening, diagnostic, or surveillance colonoscopy.

7. Study Design

7.1. Study Structure

This study is a prospective, multi-center, parallel-assigned, two-arm, randomized controlled trial. Eligible subjects will be randomly assigned into CC or SC groups in a 1:1 ratio.

7.2. Study Population

7.2.1. Inclusion criteria

(1) Age of ≥ 40 years and < 80 years
(2) Subjects who have given a signed informed consent form
(3) Informed consensus has been obtained that endoscopic resection should be performed if a lesion is found
(4) Subjects who are scheduled for screening or diagnostic colonoscopy for colorectal cancer (CRC) or surveillance colonoscopy for post-polypectomy follow-up

7.2.2. Exclusion criteria

(1) Subjects with any of the following prior history or current conditions:
  a. Contraindications to colonoscopy
  b. Inflammatory bowel disease (IBD)
  c. Colorectal cancer (CRC)
  d. Familial adenomatous polyposis (FAP)
  e. Colonic stenosis
  f. Severe organ failure (cirrhosis of Child C, heart failure of ACC / AHA stage D)
  g. Active gastrointestinal (GI) Bleeding
  h. Pregnancy
  i. Prior colorectal surgery, including colonic or rectal resection (except for appendectomy, surgery on the anus, and polypectomy)
  j. Undergo colonoscopy within 3 years
(2) Subjects with any of the following conditions per the investigator's judgement:
  a. High suspicion of IBD, CRC, and FAP.
  b. High risk of bleeding after endoscopic treatment, and difficult management of anticoagulation or antiplatelet medication.

7.3. Study Procedures

7.3.1. Screening (Screening Visit)

(1) Subjects who are scheduled for either screening colonoscopy, diagnostic

colonoscopy, or surveillance colonoscopy) due to prior history of colon polyps found during colonoscopy done 3 or more than 3 years previously will be asked if they are interested in participating in the study.

(2) Subjects who are interested will be explained the study details and provided with the informed consent form (ICF).

(3) After clear understanding of the study and signing the ICF, subjects will be checked for eligibility (inclusion and exclusion criteria), which will be recorded on the case report forms (CRFs).

7.3.2. Enrollment

Eligible subjects based on the inclusion and exclusion criteria will be enrolled in this study and relevant information will be recorded on the CRF.

7.3.3. Randomization

Before colonoscopy initiation, eligible subjects will be randomized in a 1:1 ratio to receive CC or SC.

Randomization will be based on a computer-generated randomization list prepared centrally by the sponsor. Randomization will be stratified by gender, age, and colonoscopy indication (screening, diagnostic, or surveillance) of subjects.

7.3.4. Blinding

The subject and the pathologist who performs the histopathological review will be blinded to the received procedure, while the operator will not be blinded to the study arm assigned to the subject. The investigator at each site will be unblinded to the assigned study arm of the subject, but will be blinded to the assigned study arm of the specimens when conducting the histopathological review

7.3.5. Colonoscopy Procedures (Intervention Visit)

(1) The subjects will undergo standard bowel preparation and routine pre-procedure examination for colonoscopy (standard of care)

(2) The colonoscopy (CC or SC) will be performed by qualified endoscopists, who are board-certified gastroenterologists or board-certified colorectal surgeons, using the endoscopy video processor specified in Section 7.5.

(3) During the colonoscopy, real-time video will be recorded.

(4) All proximal polyps, and polyps $\geq$ 0.6 cm found at sigmoid, rectosigmoid junction, and rectum will be resected for histopathological examination.

7.3.6. Histopathology

The resected polyps will be submitted for histologic examination by one expert pathologist at each site. Before the trial starts, a consensus meeting will be held for all participating pathologists to standardize the examination procedure and to gain consensus on the classification criteria. If any uncertainty or doubt exists, an expert in gastrointestinal pathology will serve as the adjudicator to perform a second review and make the final decision. All of the pathologists (including the final adjudicator) who will perform the histopathologic examinations and produce the pathology assessment will be blinded to the assigned study arm of the specimens. The pathology reports of the subjects will be kept securely at each site as the source data.

7.3.7. Withdrawal of Subjects

Participation in the study is completely voluntary, thus subjects can withdraw at any time during or after the procedure. Written consent will be obtained from all subjects. Withdrawing from the procedure has no negative implication on their colonoscopy examination and subsequent clinical care. A subject may be withdrawn from the research at the discretion of the endoscopist or the investigator.
Subjects who meet any of the following withdrawal criteria may be withdrawn from the study.

Withdrawal criteria:
(1) Subject who withdraws consent.
(2) Poor bowel preparation based on the four-grade modified Aronchick Bowel Preparation Scale.
(3) There is a clinical suspicion for hereditary polyposis syndrome or new case of IBD based on the colonoscopy per the investigator’s judgement.
(4) The colonoscopy procedure cannot be completed, either due to intolerance, stenosis, obstruction, huge occupying lesions, or solid stool.
(5) The colonoscopy procedure is terminated due to any procedure-related complications.
(6) Subjects with difficult anatomy or post-surgical anatomy preventing completion of the procedure using a standard colonoscope.

7.4. Endpoints

7.4.1. Primary Endpoint

The primary endpoint is the ADR of the CC as compared with that of the SC. The ADR is defined as the percentage of subjects undergoing a complete colonoscopy, who have at least one histologically confirmed adenoma detected and removed.

7.4.2. Secondary Endpoints

Secondary endpoints are listed and defined as follows:

- Polyp detection rate (PDR):
    - Percentage of subjects undergoing a complete colonoscopy who have at least one polyp detected.
- Adenomas per colonoscopy (APC):
    - Number of adenomas per colonoscopy, calculated by dividing the total number of adenomas detected by the total number of colonoscopies.
- Polyps per colonoscopy (PPC):
    - Number of polyps of any kind (i.e., adenoma, sessile serrated polyp, hyperplastic polyp, or non-neoplastic polyp) per colonoscopy, calculated by dividing the total number of polyps detected by the total number of colonoscopies.
- Non-neoplastic polypectomy rate (NNPR)
    - Percentage of subjects with at least one non-neoplastic polypectomy at the time of colonoscopy.
    - Non-neoplastic polypectomy (NNP) includes all biopsies or snare polypectomies of lesions on colonoscopy that yield non-adenomatous, non-serrated, and non-hyperplastic tissue upon histologic review.
- Sessile serrated lesions per colonoscopy (SPC):
    - Number of sessile serrated lesions (SSLs) per colonoscopy, calculated by dividing the total number of sessile serrated lesions detected by the total number of colonoscopies.
    - SSLs are not counted into the ADR as recommended by the American Society for Gastrointestinal Endoscopy/American College of Gastroenterology Task Force on Quality in Endoscopy.
- Advanced adenomas per colonoscopy (AAPC):
    - Number of advanced adenomas per colonoscopy, calculated by dividing the total number of advanced adenomas detected by the total number of colonoscopies.
    - Advanced adenomas are defined as adenomas with any of the following features present: high-grade dysplasia, villous features, or endoscopic size ≥ 1 cm. Villous features were defined as 25% or more of the composition of the polyp.
- Withdrawal time:
    - The time spent in inspecting the colonic mucosa as the endoscope is withdrawn during a colonoscopy.

7.5. Data Collection

The following subject data and relevant information will be collected and recorded using the CRF for each enrolled subject.

(1) Patient characteristics
   a. Age
   b. Gender
   c. Race
   d. Height
   e. Weight
   f. BMI
   g. Medical history of CRC from any first degree relatives (including parents, siblings, and children)
   h. Smoking

(2) Disease-related characteristics
   a. Fecal immunochemical test (FIT) results
   b. Colonoscopy indication

(3) Procedure-related characteristics
   a. Bowel preparation quality (based on the four-grade modified Aronchick Bowel Preparation Scale) (Hsu et al., 2016): excellent (1), good (2), fair (3), poor (4)
   b. Experience of the operator /endoscopists
   c. Cecal intubation time (min and sec)
   d. Withdrawal time (min and sec)
   e. Effective procedure time (min and sec)
   f. Endoscopy video processor models

(4) Lesion characteristics
   a. Size
   b. Location
   c. Morphology

(5) Study outcomes
   a. Number of polyps detected
   b. Number of adenomas detected
   c. Number of sessile serrated lesions
   d. Number of advanced adenomas
   e. Number of non-neoplastic resections

8. Statistical Analysis

8.1. Sample Size Estimation

The sample size is calculated based on the evaluation of primary outcome ADR. Based on the results of meta-analysis, an expected ADR ranges from around 20% to 40% for AI-aided colonoscopy and SC (Barua et al., 2021; Hassan et al., 2021; Mohan et al., 2020; Shaukat et al., 2021; Spadaccini et al., 2021). A sample size ranging from 198 to 297 per arm is required to establish non-inferiority with a non-inferiority margin of 10%, power of 80% and an alpha level of 2.5% (one-sided). Considering a withdrawal rate of 10% due to potential exclusions and drop-outs, the enrollment goal is 220 to 330 subjects per arm.

However, interim analysis of this study in March 2023 showed that the ADR in our SC group is 49%, which is significantly higher than the ADR results acquired from meta-analysis (around 20%); therefore, the enrollment goal is increased to 750 subjects per arm. (i.e., 1500 subjects in total.)

8.2. Analysis Population

Subjects who complete the CC or SC procedure and do not meet any of the withdrawal criteria will be included into the analysis population for statistical analysis.

8.3. Statistical Methods

The primary analysis will be the comparison of ADR between CC and SC. Differences in ADR between two arms will be expressed as relative risk (RR) with 95% CIs. Non-inferiority will be met for the primary endpoint (ADR) if the lower 2-sided 95% confidence interval (CI) excludes a 10% or greater difference in favor of the control group. The 10% non-inferiority margin reflects a typical maximum clinically acceptable difference for comparative studies.

If non-inferiority is demonstrated for the primary endpoint, the endpoint will be assessed for superiority (one-sided p value <0.025) using the Fisher exact test.

Categorical variables will be described by frequency counts and percentages. Quantitative variables will be described by mean and standard deviations. Chi-square and t-tests will be used to compare categorical and continuous variables between the two groups, respectively.

ADR and PDR will be stratified by subject characteristics (including age, gender, and colonoscopy indication) and experience of the operator; APC and PPC will be stratified by lesion characteristics (including size, location, and morphology of each polyp).

A p value <0.05 will be considered statistically significant.

All statistical analyses will be performed using R or Python software.

9. Risks and Benefits

Risks and Discomforts

The risk of perforation of the colon during screening/surveillance colonoscopy with polypectomy is 1:1000. There is risk of bleeding following polypectomy, but bleeding is readily fixable. These risks would apply to the subjects regardless of whether they choose to participate in the study or not. It is not anticipated that participation in this study will directly pose any psychological risks to the subject.

Potential Benefits

Confirmation of the clinical efficacy of the AI-aided colonoscopy may aid future subjects who undergo screening or surveillance colonoscopy to identify precancerous lesions and further prevent colorectal cancer.

10. Ethical Compliance

Signed written informed consent forms will be obtained from all participating subjects before enrollment.

11. References

Barua, I., Vinsard, D. G., Jodal, H. C., Løberg, M., Kalager, M., Holme, Ø., Misawa, M., Bretthauer, M., & Mori, Y. (2021). Artificial intelligence for polyp detection during colonoscopy: a systematic review and meta-analysis. *Endoscopy*, *53*(3), 277-284. https://doi.org/10.1055/a-1201-7165

Endoscopic Classification Review, G. (2005). Update on the paris classification of superficial neoplastic lesions in the digestive tract. *Endoscopy*, *37*(6), 570-578. https://doi.org/10.1055/s-2005-861352

Hassan, C., Spadaccini, M., Iannone, A., Maselli, R., Jovani, M., Chandrasekar, V. T., Antonelli, G., Yu, H., Areia, M., Dinis-Ribeiro, M., Bhandari, P., Sharma, P., Rex, D. K., Rösch, T., Wallace, M., & Repici, A. (2021). Performance of artificial intelligence in colonoscopy for adenoma and polyp detection: a systematic review and meta-analysis. *Gastrointestinal Endoscopy*, *93*(1), 77-85.e76. https://doi.org/10.1016/j.gie.2020.06.059

Hsu, W.-F., Liang, C.-C., Lin, C.-K., Lee, T.-H., & Chung, C.-S. (2016). A modified bowel preparation protocol improves the quality of bowel cleansing for colonoscopy. *Advances in Digestive Medicine*, *3*(3), 144-147. https://doi.org/10.1016/j.aidm.2015.12.001

Mohan, B. P., Facciorusso, A., Khan, S. R., Chandan, S., Kassab, L. L., Gkolfakis, P., Tziatzios, G., Triantafyllou, K., & Adler, D. G. (2020). Real-time computer aided colonoscopy versus standard colonoscopy for improving adenoma detection rate: A meta-analysis of randomized-controlled trials. *EClinicalMedicine*, *29-30*, 100622. https://doi.org/10.1016/j.eclinm.2020.100622

Shaukat, A., Kahi, C. J., Burke, C. A., Rabeneck, L., Sauer, B. G., & Rex, D. K. (2021). ACG Clinical Guidelines: Colorectal Cancer Screening 2021. *Official journal of the American College of Gastroenterology | ACG*, *116*(3), 458-479. https://doi.org/10.14309/ajg.0000000000001122

Spadaccini, M., Iannone, A., Maselli, R., Badalamenti, M., Desai, M., Chandrasekar, V. T., Patel, H. K., Fugazza, A., Pellegatta, G., Galtieri, P. A., Lollo, G., Carrara, S., Anderloni, A., Rex, D. K., Savevski, V., Wallace, M. B., Bhandari, P., Roesch, T., Gralnek, I. M., Sharma, P., Hassan, C., & Repici, A. (2021). Computer-aided detection versus advanced imaging for detection of colorectal neoplasia: a systematic review and network meta-analysis. *The Lancet. Gastroenterology & Hepatology*, *6*(10), 793-802. https://doi.org/10.1016/S2468-1253(21)00215-6

12. Appendices

12.1. Bowel preparation scale (Hsu et al., 2016)

12.2. Paris classification (Endoscopic Classification Review, 2005)
