# Supplementary material for: Computer-Assisted Colonoscopy in High–Adenoma Detection Rate Settings in a High-Risk Population: A Randomized Clinical Trial
Source: JAMA Netw Open. 2026 Apr 15;9(4):e264881. doi: 10.1001/jamanetworkopen.2026.4881 (PMC13084460; doi:10.1001/jamanetworkopen.2026.4881)
Supplement: Supplement 2. — eMethods 1. Detailed Description of the AI System eMethods 2. Definitions of Secondary End Points eFigure. Adjusted IRR for Adenomas per Colonoscopy With CAD vs Standard Colonoscopy eTable 1. Comparison of the Sessile Serrated Lesion Detection Rate (SSLDR) Between CAD-Assisted and Standard Colonoscopy eTable 2. Regression Analyses for Factors Associated With Adenoma Detection Rate (ADR) for FIT-Positive Colonoscopy eTable 3. Comparison of Adenoma Detection Rate in the Standard Colonoscopy Group Between the First and Second Halves of the Study Period eTable 4. Comparison of Adenomas Detected per Colonoscopy by Lesion Characteristics Between CAD-Assisted and Standard Colonoscopy eTable 5. Comparison of Adenomas Detected per Colonoscopy Among FIT-Positive Patients: CAD-Assisted vs Standard Colonoscopy [file jamanetwopen-e264881-s002.pdf]

# Supplementary Online Content

Hsu WF, Kuo CY, Yen HH, et al. Computer-assisted colonoscopy in high–adenoma detection rate settings in a high-risk population: a randomized clinical trial. *JAMA Netw Open.* 2026;9(4):e264881. doi:10.1001/jamanetworkopen.2026.4881

**eMethods 1.** Detailed Description of the AI System
**eMethods 2.** Definitions of Secondary End Points
**eFigure.** Adjusted IRR for Adenomas per Colonoscopy With CAD vs Standard Colonoscopy
**eTable 1.** Comparison of the Sessile Serrated Lesion Detection Rate (SSLDR) Between CAD-Assisted and Standard Colonoscopy
**eTable 2.** Regression Analyses for Factors Associated With Adenoma Detection Rate (ADR) for FIT-Positive Colonoscopy
**eTable 3.** Comparison of Adenoma Detection Rate in the Standard Colonoscopy Group Between the First and Second Halves of the Study Period
**eTable 4.** Comparison of Adenomas Detected per Colonoscopy by Lesion Characteristics Between CAD-Assisted and Standard Colonoscopy
**eTable 5.** Comparison of Adenomas Detected per Colonoscopy Among FIT-Positive Patients: CAD-Assisted vs Standard Colonoscopy

This supplemental material has been provided by the authors to give readers additional information about their work.

© 2026 Hsu WF et al. *JAMA Network Open*.

**eMethods 1.** Detailed Description of the AI System

**Algorithm and Architecture**

The computer-Aided Detection (CAD) system used in this study is aetherAI Endo™ (aetherAI Co., Ltd., Taiwan), which has received approval from the Taiwan Food and Drug Administration (TFDA License No. 007508). The core algorithm employs a YOLO (You Only Look Once) single-stage object detection model, chosen for its high inference speed and suitability for real-time clinical applications. The model takes endoscopic video frames as input and predicts bounding boxes with associated confidence scores for detected polyps. The network was initialized with weights pre-trained on the MSCOCO dataset and fine-tuned using the Distance-IoU (CIOU) loss function to optimize bounding box regression.

**Training and Development Datasets**

The model was trained on a large-scale dataset comprising 172,933 colonoscopy images collected from 3,194 patients across two medical centers in Taiwan: Cathay General Hospital (163,423 images from 3,122 patients) and National Taiwan University Hospital (9,510 images from 72 patients). The training dataset included diverse polyp types and non-polyp structures to ensure generalizability. Data augmentation techniques, including random flips, color jittering, random rotations, and the Mosaic method, were applied during training to enhance model robustness. Images were acquired using high-definition processors (Olympus EVIS LUCERA ELITE CV-290/CV-260 and Fujifilm VP-7000).

**User Interface and Interaction**

The system operates in real-time. When a potential lesion is detected, the

© 2026 Hsu WF et al. *JAMA Network Open*.

system displays a visual bounding box around the lesion on a dedicated monitor adjacent to the main endoscopic view. This visual cue alerts the endoscopist to inspect the area more closely. The endoscopist serves as the final decision-maker; the system does not automatically classify or characterize the lesion histologically but serves solely as a detection aid.

**Performance Validation**

Prior to the clinical trial, the model was validated on four independent datasets from the participating hospitals, achieving high per-frame sensitivity (range: 90.62%–97.95%) and specificity (range: 93.85%–97.17%).

© 2026 Hsu WF et al. *JAMA Network Open*.

**eMethods 2.** Definitions of Secondary End Points

Secondary end points reported in the intention-to-treat population (Table 2) were defined as follows:

- Adenomas per Colonoscopy (APC): Calculated as the total number of adenomas detected divided by the total number of colonoscopies performed in each group.
- Polyps per Colonoscopy (PPC): Calculated as the total count of all detected polyps (including adenomas, sessile serrated lesions, hyperplastic polyps, and non-neoplastic polyps) divided by the total number of colonoscopies.
- Advanced Adenomas per Colonoscopy (AAPC): Calculated as the total number of advanced adenomas detected divided by the total number of colonoscopies. Advanced adenomas were defined as adenomas presenting with any of the following features: high-grade dysplasia, villous histology, or an endoscopic size ≥ 10 mm.
- Non-Neoplastic Polypectomy Rate (NNPR): Defined as the percentage of patients undergoing at least one resection (biopsy or snare polypectomy) of a lesion that was confirmed to be non-neoplastic (i.e., non-adenomatous, non-serrated, and non-hyperplastic) upon histologic review.
- Sessile Serrated Lesion Detection Rate (SSLDR): Defined as the percentage of patients with at least one histologically confirmed sessile serrated lesion (SSL).
- Withdrawal Time: Defined as the time elapsed from cecal intubation to the removal of the colonoscope from the anus. In cases where biopsies

© 2026 Hsu WF et al. *JAMA Network Open*.

or polypectomies were performed, the time dedicated to these therapeutic procedures was subtracted to calculate the clean withdrawal time (inspection time).

© 2026 Hsu WF et al. *JAMA Network Open*.

**eFigure.** Adjusted IRR for Adenomas per Colonoscopy With CAD vs Standard Colonoscopy

| | Adjusted IRR (95% CI) | P value |
|---|---|---|
| Overall | 1.16 (1.00-1.34) | 0.05 |
| FIT positive | 1.18 (1.00-1.38) | 0.05 |
| Colonoscopy screening | 0.69 (0.43-1.10) | 0.12 |
| Surveillance | 1.27 (0.83-1.95) | 0.28 |
| Symptomatic | 1.18 (0.79-1.78) | 0.42 |
| Junior endoscopists | 1.33 (1.01-1.75) | 0.05 |
| Senior endoscopists | 1.12 (0.95-1.31) | 0.17 |

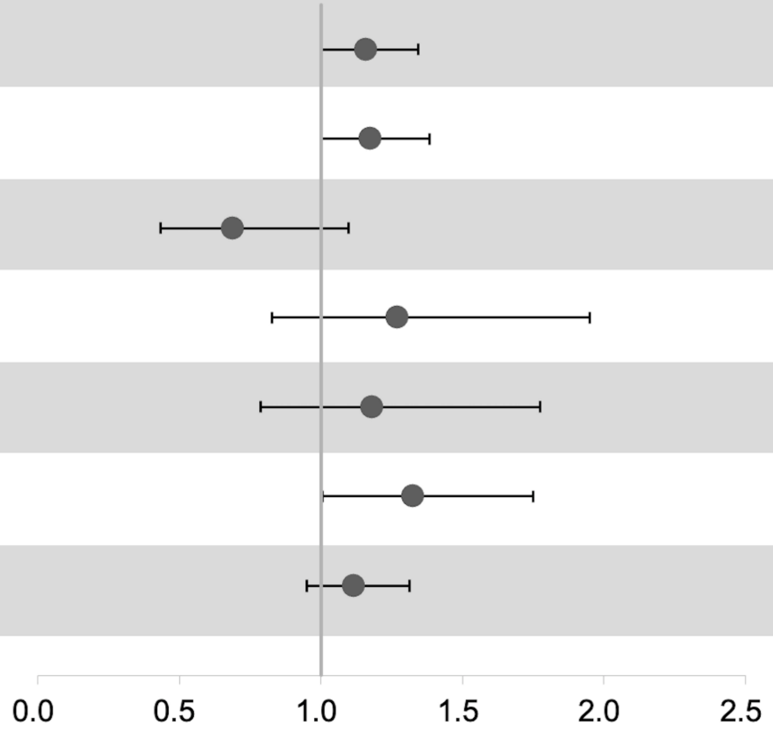


CAD, computer-aided detection; CI, confidence interval; FIT, fecal immunochemical test; IRR, incidence rate ratios

© 2026 Hsu WF et al. *JAMA Network Open*.

**eTable 1.** Comparison of the Sessile Serrated Lesion Detection Rate (SSLDR) Between CAD-Assisted and Standard Colonoscopy

| | **Standard colonoscopy, %** | **CAD-assisted colonoscopy, %** | ***p-value*** |
|---|---|---|---|
| Overall | 4.8 | 4.3 | 0.63 |
| Indication for colonoscopy | | | |
| FIT-positive | 3.5 | 3.2 | 0.76 |
| Colonoscopy screening | 10.8 | 2.9 | 0.10 |
| Surveillance | 6.2 | 11.7 | 0.22 |
| Symptomatic | 4.9 | 4.6 | 1 |
| Endoscopist experience, n (%) | | | |
| Senior endoscopist (≥ 2,000 procedures) | 4.1 | 3.7 | 0.74 |
| Junior endoscopist (<2,000 procedures) | 6.8 | 5.7 | 0.64 |

CAD=computer-aided detection

© 2026 Hsu WF et al. *JAMA Network Open*.

**eTable 2.** Regression Analyses for Factors Associated With Adenoma Detection Rate (ADR) for FIT-Positive Colonoscopy

| | **Univariate analysis** | | ***Multivariable analysis** | |
|---|---|---|---|---|
| | **OR (95% CI)** | ***p* value** | **aOR (95% CI)** | ***p* value** |
| Age | 1.04 (1.03-1.06) | <.0001 | 1.04 (1.02-1.06) | <.0001 |
| Male vs. female | 1.95 (1.47-2.58) | <.0001 | 1.90 (1.43-2.54) | <.0001 |
| Senior endoscopist vs. junior endoscopist | 1.22 (0.91-1.63) | 0.19 | | |
| CAD-assisted colonoscopy vs. standard colonoscopy | 1.40 (1.06-1.84) | 0.02 | 1.39 (1.05-1.86) | 0.02 |

*Adjusted for participating endoscopy centers

aOR, adjusted odds ratio; CAD, computer-aided detection; CI, confidence interval; FIT, fecal immunochemical test; OR, odds ratio.

© 2026 Hsu WF et al. *JAMA Network Open*.

**eTable 3.** Comparison of Adenoma Detection Rate in the Standard Colonoscopy Group Between the First and Second Halves of the Study Period

| Period | Total Procedures (N) | Adenoma Detection Rate, n. (%) | P Value |
|---|---|---|---|
| First Half (Early Phase) | 340 | 180 (52.9%) | 0.34 |
| Second Half (Late Phase) | 341 | 183 (53.7%) | |

P value was calculated using the Chi-square test. This analysis was performed to assess potential learning effects or contamination bias.

© 2026 Hsu WF et al. *JAMA Network Open*.

**eTable 4.** Comparison of Adenomas Detected per Colonoscopy by Lesion Characteristics Between CAD-Assisted and Standard Colonoscopy

| | Study arms | | Comparison of CAD-assisted colonoscopy vs. standard colonoscopy | | |
|---|---|---|---|---|---|
| | CAD-assisted colonoscopy | Standard colonoscopy | | | |
| **Adenomas per colonoscopy** | **Mean (SD)** | **Mean (SD)** | **Adjusted MD* (95% CI)** | **Adjusted IRR* (95% CI)** | **p-value** |
| **Adenoma size** | | | | | |
| <=5 | 0.91 (1.46) | 0.75 (1.27) | 0.16 (0.01- 0.31) | 1.22 (1.03-1.43) | 0.02 |
| 6-9 | 0.31 (0.74) | 0.29 (0.82) | 0.02 (-0.06-0.11) | 1.13 (0.87-1.46) | 0.37 |
| >=10 | 0.19 (0.50) | 0.17 (0.45) | 0.02 (-0.03-0.07) | 1.13 (0.85-1.49) | 0.40 |
| **Location** | | | | | |
| Proximal colon | 0.79 (1.33) | 0.67 (1.26) | 0.12 (-0.02-0.26) | 1.20 (1.01-1.44) | 0.04 |
| Distal colon | 0.62 (0.97) | 0.54 (0.96) | 0.08 (-0.02-0.18) | 1.16 (0.97-1.37) | 0.10 |

© 2026 Hsu WF et al. *JAMA Network Open*.

| | | | | | |
|---|---|---|---|---|---|
| **Morphology** | | | | | |
| Polypoid | 0.73 (1.38) | 0.65 (1.31) | 0.09 (-0.06-0.23) | 1.15 (0.95-1.39) | 0.15 |
| Non-polypoid | 0.66 (1.31) | 0.55 (1.17) | 0.11 (-0.02-0.25) | 1.22 (0.99-1.49) | 0.06 |

CAD=computer-aided detection. MD=mean difference. IRR=incidence rate ratio. PD=proportion difference. OR=odds ratio. * MD, OR, and IRR were adjusted for all randomization stratification variables.

© 2026 Hsu WF et al. *JAMA Network Open*.

**eTable 5.** Comparison of Adenomas Detected per Colonoscopy Among FIT-Positive Patients: CAD-Assisted vs Standard Colonoscopy

| | **Study arms** | | **Comparison of CAD-assisted colonoscopy vs. standard colonoscopy** | | |
|---|---|---|---|---|---|
| | **CAD-assisted colonoscopy** | **Standard colonoscopy** | | | |
| **Adenomas per colonoscopy** | **Mean (SD)** | **Mean (SD)** | **Adjusted MD* (95% CI)** | **Adjusted IRR* (95% CI)** | **p-value** |
| **Adenoma size** | | | | | |
| <=5 | 1.05 (1.59) | 0.84 (1.36) | 0.22 (0.02-0.41) | 1.26 (1.03-1.53) | 0.02 |
| 6-9 | 0.35 (0.83) | 0.34 (0.94) | 0.01 (-0.11-0.13) | 1.07 (0.78-1.47) | 0.67 |
| >=10 | 0.23 (0.54) | 0.21 (0.49) | 0.02 (-0.05-0.09) | 1.10 (0.81-1.50) | 0.53 |
| **Location** | | | | | |
| Proximal colon | 0.91 (1.44) | 0.76 (1.40) | 0.15 (-0.04-0.34) | 1.21 (0.98-1.49) | 0.08 |
| Distal colon | 0.72 (1.04) | 0.62 (1.05) | 0.10 (-0.04-0.24) | 1.16 (0.95-1.41) | 0.15 |

© 2026 Hsu WF et al. *JAMA Network Open*.

| | | | | | |
|---|---|---|---|---|---|
| **Morphology** | | | | | |
| Polypoid | 0.86 (1.50) | 0.74 (1.44) | 0.12 (-0.08-0.32) | 1.16 (0.93-1.45) | 0.18 |
| Non-polypoid | 0.76 (1.41) | 0.64 (1.32) | 0.13 (-0.06-0.31) | 1.21 (0.95-1.55) | 0.12 |

CAD=computer-aided detection. MD=mean difference. IRR=incidence rate ratio. PD=proportion difference. OR=odds ratio. * MD, OR, and IRR were adjusted for all randomization stratification variables.

© 2026 Hsu WF et al. *JAMA Network Open*.
