# Supplementary material for: Computer-Assisted Colonoscopy in High–Adenoma Detection Rate Settings in a High-Risk Population: A Randomized Clinical Trial
Source: JAMA Netw Open. 2026 Apr 15;9(4):e264881. doi: 10.1001/jamanetworkopen.2026.4881 (PMC13084460; doi:10.1001/jamanetworkopen.2026.4881)
Supplement: Supplement 3. — Data Sharing Statement [file jamanetwopen-e264881-s003.pdf]

Data Sharing Statement

Hsu. Computer-Assisted Colonoscopy in High–Adenoma Detection Rate Settings in a High-Risk Population. *JAMA Netw Open*. Published April 15, 2026.
doi:10.1001/jamanetworkopen.2026.4881

Data
Additional Information: ClinicalTrials.gov (NCT03842059) URL:
https://clinicaltrials.gov/study/NCT03842059
Data available: No
